# Natural History of Nipah Virus in Hamsters: Strain, Route, and Sex-Associated Variability Characterized Using Large Datasets to Inform Pre-Clinical Study Design

**DOI:** 10.1093/infdis/jiaf549

**Published:** 2025-10-29

**Authors:** Katherine A Davies, Stephen R Welch, JoAnn D Coleman-McCray, Teresa E Sorvillo, Virginia Aida-Ficken, Christina F Spiropoulou, Jessica R Spengler

**Affiliations:** Viral Special Pathogens Branch, Division of High-Consequence Pathogens and Pathology, Centers for Disease Control and Prevention, Atlanta, Georgia, USA; United States Department of Agriculture, Zoonotic and Emerging Disease Research Unit, National Bio and Agro-Defense Facility, Agricultural Research Service, Manhattan, Kansas, USA; Viral Special Pathogens Branch, Division of High-Consequence Pathogens and Pathology, Centers for Disease Control and Prevention, Atlanta, Georgia, USA; Viral Special Pathogens Branch, Division of High-Consequence Pathogens and Pathology, Centers for Disease Control and Prevention, Atlanta, Georgia, USA; Viral Special Pathogens Branch, Division of High-Consequence Pathogens and Pathology, Centers for Disease Control and Prevention, Atlanta, Georgia, USA; Infectious Disease Department, CDC Foundation, Atlanta, Georgia, USA; Viral Special Pathogens Branch, Division of High-Consequence Pathogens and Pathology, Centers for Disease Control and Prevention, Atlanta, Georgia, USA; Department of Pathobiology, College of Veterinary Medicine, Auburn University, Auburn, Alabama, USA; Viral Special Pathogens Branch, Division of High-Consequence Pathogens and Pathology, Centers for Disease Control and Prevention, Atlanta, Georgia, USA; Viral Special Pathogens Branch, Division of High-Consequence Pathogens and Pathology, Centers for Disease Control and Prevention, Atlanta, Georgia, USA

## Abstract

Nipah virus (NiV) comprises two strains, Malaysia and Bangladesh, associated with severe respiratory and/or neurological disease in humans. Experimentally infected Syrian hamsters demonstrate the full spectrum of clinical signs reported in humans, serving as valuable pre-clinical screening models for NiV disease. Medical countermeasure development relies on well-characterized disease models to understand disease progression, guiding pre-clinical and clinical trial design. Variability in NiV-disease presentation and outcome necessitates large group sizes in animal model natural history studies. To advance the use of hamsters in NiV pre-clinical studies, we analyzed in-house data from 19 independent studies comprising over 500 hamsters intranasally or intraperitoneally infected with NiV-Malaysia or NiV-Bangladesh. We demonstrate strain- and route-associated differences in clinical course, lethality, and viral loads, presenting cohort and individual data. These analyses provide key data to guide experimental design for pathogenesis, pathophysiology, and medical countermeasure studies.

## Teaser

Hamsters model NiV disease, recapitulating key aspects of human illness. Disease course and outcomes vary with virus strain, route, and dose. Analyses of over 500 animals provide essential data to guide experimental design in natural history and countermeasure screening studies.

Nipah virus (NiV; *Paramyxoviridae*; Henipavirus) causes severe, often fatal disease in humans. NiV infection generally presents with non-specific signs (fever, malaise) progressing to severe acute respiratory decompensation and/or neurological disease; patients can also exhibit mild signs or remain asymptomatic but present years later as neurological relapse. Since the first described outbreak in Malaysia and Singapore (1998–1999), more than 50 independent outbreaks have been documented in India, Bangladesh, and the Philippines [1]. There are two recognized strains of NiV, the Malaysia strain (NiV-M), associated with the initial outbreak and a subsequent outbreak in the Philippines in 2014, and the Bangladesh strain (NiV-B), responsible for all other known outbreaks [1]. Currently, no Food and Drug Administration (FDA)-approved medical countermeasures (MCM) are available for NiV infections.

For highly pathogenic viruses like NiV, which have had relatively low incidence to date, human MCM trials are often hindered by delays in outbreak detection, small case numbers, and limited person-to-person transmission [2]. MCMs can be approved through alternate regulatory pathways such as the FDA Animal Rule, which typically requires the use of qualified animal models and demonstration of safety and efficacy in more than one species [3]. Rodents offer several advantages for preclinical studies, including low costs and ease of handling. While mice are susceptible to NiV infection, they do not develop clinical signs unless immunocompromised [4, 5]. In contrast, Syrian hamsters can develop disease following infection without the need for immunosuppression or species adaptation. Key aspects of NiV disease in humans, both respiratory and neurological, are well recapitulated in hamsters [6]. However, the variable disease spectrum in this model poses challenges for study design and interpretation. Disease course and lethality can differ significantly depending on experimental parameters such as virus strain, dose, and route of inoculation, resulting in considerable interstudy variability. Additionally, data are often reported at the group level rather than the individual level, and small sample sizes, necessitated by biosafety level 4 (BSL-4) constraints, further limit comparability. These limitations highlight the need for comprehensive aggregate data analyses to inform and optimize pre-clinical study design.

To better understand the progression of NiV disease in Syrian hamsters over time and further validate the model, we analyzed aggregate data collected between 2018 and 2025 from 19 independent studies comprising over 500 animals. We investigated clinical presentation, outcomes, and tissue distribution in hamsters infected with NiV-M or NiV-B via intranasal (IN) or intraperitoneal (IP) inoculation, with the aim of establishing a robust foundation for pre-clinical study design and interpretation. This report represents the largest aggregate dataset of experimentally infected Syrian hamsters with NiV to date, providing critical insights to support drug and vaccine development, particularly in selecting the most representative animal model, determining optimal timing for intervention, and identifying meaningful clinical endpoints.

## METHODS

### Biosafety

Work with infectious viruses or infected animals was conducted in a BSL-4 laboratory at the U.S. Centers for Disease Control and Prevention (CDC). Animal studies were approved by the CDC Institutional Animal Care and Use Committee (#2798, 2928, 2956, 3220, 3419). A subset of these studies was also approved by the United States Army Medical Research and Development Command Animal Care and Use Review Office. Work was performed in an AAALAC International-accredited facility and conducted in accordance with the *Guide for the Care and Use of Laboratory Animals*.

### Viruses

NiV-M (GenBank accession no. AF212302 [7], Virharv #813744), obtained from a human cerebrum sample, was isolated in Vero E6 cells and passaged once in Vero cells. NiV-B (GenBank accession no. AY988601 [8], Virharv #813747), obtained from a human throat swab, was isolated in Vero E6 cells and passaged once in Vero cells. All viral stocks were verified by next-generation sequencing and confirmed to be mycoplasma-free.

### Animal Studies

**Figure 1.**
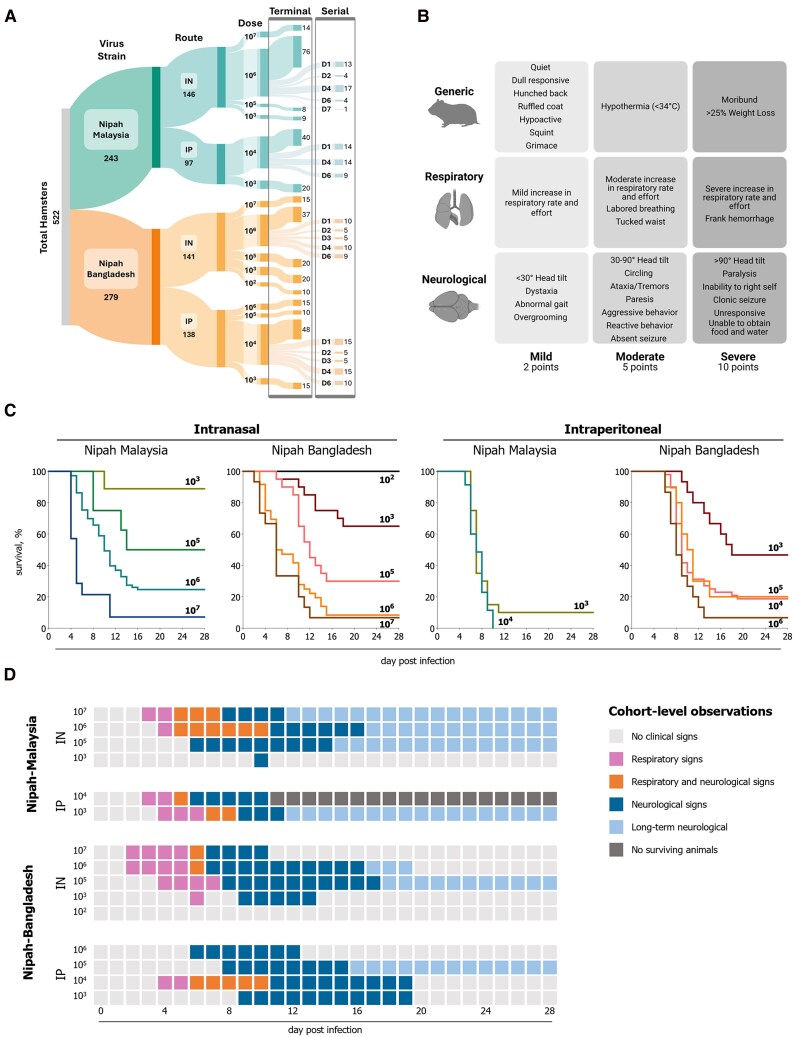
Nipah virus (NiV) inoculation strain and dose alter clinical progression and outcome in Syrian hamsters. *A*, Graphic illustration (Sankey diagram) of cohort group sizes used for data analyses. Experimental cohorts are defined as Syrian hamsters inoculated with NiV Malaysia or NiV Bangladesh intranasally [IN] or intraperitoneally [IP]. Terminal groups indicate those meeting the terminal endpoint or surviving to the study end. These cohorts were used for clinical course analyses and assessment of tissue viral RNA levels. Terminal animals used for outcome analyses were only those originally defined as terminal animals in the study plan and excluded any animals originally intended for serial sacrifice but met criteria prior to the pre-determined time point to prevent bias in lethality values. Serial animals were euthanized at pre-determined time points (1–7 days post-infection) and were used for the assessment of tissue viral RNA levels. *B*, Clinical scoring matrix for Nipah virus-infected Syrian hamsters. Mild (2 points), moderate (5 points), and severe (10 points) signs are indicated as either generic, respiratory, or neurological. Scores are additive, with any animals scoring ≥10 points meeting euthanasia criteria. Graphic generated using BioRender. *C*, Kaplan–Meier survival curves of Syrian hamsters inoculated with NiV Malaysia (NiV-M) or NiV Bangladesh (NiV-B) intranasally (IN) or intraperitoneally (IP). Dose range between 10^2^–10^7^ TCID_50_. For survival analysis, the numbers included in each cohort are NiV-M IN 10^3^ [n = 9], 10^5^ [n = 9], 10^6^ [n = 73], 10^7^ [n = 9]; NiV-B IN 10^2^ [n = 10], 10^3^ [n = 20], 10^5^ [n = 20], 10^6^ [n = 36], 10^7^ [n = 15]; NiV-M IP 10^3^ [n = 20], 10^4^ [n = 35]; NiV-B IP 10^3^ [n = 15], 10^4^ [n = 48], 10^5^ [n = 10], 10^6^ [n = 15]. *D*, Cohort-level observations of respiratory and/or neurological presentation in Syrian hamsters inoculated with NiV. Dose range between 10^2^–10^7^ TCID_50_. IN, intranasal route; IP, intraperitoneal route. Observations of clinical signs observed at a cohort-level are indicated by day: ≥ 1 individual exhibiting only respiratory signs (pink), ≥ 1 individual exhibiting only neurological signs (blue), both respiratory and neurological signs observed within the experimental cohort (i.e., by separate individuals or concurrently in an individual; orange), survivors with ongoing neurological signs after last neurological associated death (light blue), no signs (light gray), no survivors (dark gray).

Data were compiled from 19 studies (2018–2025), comprising 522 NiV-infected and 64 mock-infected Syrian hamsters (Envigo, HsdHan:Aura; 8902F/8903F/8902M/8903M) (Figure 1*A*). NiV-infected animals were unvaccinated or untreated controls from unpublished and previously published studies [9–14]. Experimental cohorts were defined by matching virus strain, dose, and inoculation route. Housing details are provided in Supplementary Methods. Hamsters were inoculated intranasally (IN; 100 µL total, divided between nares; target doses: 10^7^, 10^6^, 10^5^, 10^3^, or 10^2^ TCID_50_) or intraperitoneally (IP; 500 or 2000 µL, split bilaterally; target doses: 10^7^, 10^6^, 10^5^, 10^4^, or 10^3^ TCID_50_) with either NiV-M or NiV-B. The delivered dose was confirmed by titration on Vero cells. Individual details (age, sex, inoculation route, and dose) can be found in Supplementary Table 1. Hamsters were assessed daily for clinical signs (Figure 1*B*), weight change, and temperature measured using subcutaneous microchip transponders (see Supplementary Materials). Euthanasia was by isoflurane overdose at pre-defined time points (serial, 1–7 days post-infection [dpi]), clinical score ≥10 points (terminal), or study endpoint (28 dpi, survivor). Animals that died on or before scheduled serial endpoint were classified as terminal for clinical and viral load analyses but excluded from survival analysis to avoid bias in lethality values.

### RNA Extraction and Quantitative PCR

RNA was extracted from blood and tissues using either the MagMAX-96 Total RNA isolation kit or the MagMAX Pathogen RNA/DNA Kit on the KingFisher Flex or KingFisher Apex systems (all Thermo Fisher Scientific). RNA was stored at −80°C before analysis. Viral RNA in tissues was quantified by reverse transcription quantitative PCR (RT-qPCR) targeting the NiV nucleoprotein (N) [15] and standardized using in-house validated reference gene RT-qPCR assays for Peptidylprolyl isomerase A (*Ppia*) and Hypoxanthine phosphoribosyl transferase (*Hprt*) [16], or using a commercial Eukaryotic 18s rRNA Endogenous Control assay (Thermo Fisher Scientific). Additional details provided in Supplementary Materials.

### Graphing and Statistical Analysis

Individual participant data (IPD) included in analyses are summarized in Supplementary Table 1. Graphs were created in GraphPad Prism (v10). Analyses in GraphPad Prism included Kaplan–Meier plots (Log-Rank [Mantel-Cox] test), Spearman correlations (clinical score vs weight or temperature), and Mann-Whitney tests (vRNA). IPD meta-analyses to evaluate interstudy variances were performed in R (v4) using the ordinal, coxme, and survival packages. Disease progression was modeled with ordinal mixed-effects (clinical score as outcome; day post-infection as fixed effect; animal and study as random effects). Survival was assessed using mixed-effects Cox models with study as a frailty term. Standard deviations (SD) of random effects indicate the level of variability.

## RESULTS

### Aggregate Data Analysis Delineates Effect of Dose and Strain on Clinical Progression and Outcome in NiV-infected Syrian Hamsters

Reports of NiV infection in hamsters show variability by virus strain and dose (Supplementary Table 2). To investigate these factors, we analyzed data from 348 unvaccinated, untreated NiV-infected hamsters monitored for up to 28 dpi. Data were aggregated from 19 independent studies using NiV-M or NiV-B inoculated IN or IP, at doses from 10^2^–10^7^ TCID_50_. Several findings were consistent with previous reports. We observed dose-dependent effects [17–21], with higher doses correlating with increased lethality, and route-dependent effects, with IP inoculation producing higher lethality than IN at equivalent doses (Figure 1*C*). NiV-M administered via the IP route resulted in the highest lethality (90%–100%) (Figure 1*C*) [9, 11, 17, 20, 22–25]. Notably, NiV-B was more lethal than NiV-M following IN inoculation (10^6^ TCID_50_, *P* = .0086), whereas the opposite was true with IP inoculation (10^4^ TCID_50_, *P* < .0001).

Both respiratory and neurological signs were observed in cohorts infected with NiV-M or NiV-B (Figure 1*D*), but variability in the incidence of respiratory and neurological signs was observed across inoculation doses and routes (Table 1). In cohorts inoculated IN, a decreasing dose was associated with a lower incidence of respiratory signs and a higher incidence of neurological signs. Clinical patterns were virus strain independent in IN-inoculated cohorts but varied more by strain in IP-inoculated cohorts. Following IP inoculation, NiV-M was more likely to cause respiratory signs than NiV-B; respiratory signs were rarely observed with NiV-B delivered IP. In IP-inoculated cohorts, the incidence of neurological signs decreased with decreasing dose, yet remained ≥ 45% across all doses evaluated. Lower doses resulted in a delayed onset of clinical signs compared to higher doses. For high-dose groups (IN- and IP-inoculated, ≥10^4^ TCID_50_), the mean day of onset (MDO) for respiratory signs was 4.8 dpi (range: 2–8), significantly different (*P* = .0002) from low-dose groups (IN- and IP-inoculated, ≤10^3^ TCID_50_), where MDO for respiratory signs was 6.6 dpi (range: 4–10). MDO of neurological signs was 8.5 dpi (range: 3–17) for high-dose groups and 10.2 dpi (range: 7–15) for low-dose groups, demonstrating statistical significance (*P* = .0015). This trend was similarly reflected in the mean-time-to-death (MTTD); lower doses were associated with an increased MTTD, indicating a temporal shift in the clinical disease profile to later days post inoculation (Table 1).

**Table 1.** Dose-dependent Outcome and Incidence of Respiratory and Neurological Signs for Nipah-inoculated Syrian Hamsters

Strain	Route	Dose TCID_50_	Number	Survival	Incidence		MDO, day			MTTD, day		
					R	N	All	R	N	All	R	N
**Nipah-Malaysia**	IN	10^7^	14	7%	93%	29%	4.7	4.4	6.0	5.5	4.6	11.0
		10^6^	73	25%	59%	74%	6.5	4.7	8.0	8.9	5.3	10.6
		10^5^	8	50%	0%	75%	8.2	…	8.2	10.8	…	10.8
		10^3^	9	89%	0%	11%	10.0	…	10.0	10.0	…	10.0
	IP	10^4^	35	0%	40%	66%	6.3	5.5	6.8	7.3	5.9	8.1
		10^3^	20	10%	55%	45%	6.6	6.1	7.5	7.4	6.7	8.6
**Nipah-Bangladesh**	IN	10^7^	15	7%	33%	27%	5.1	3.8	7.4	6.3	4.6	9.8
		10^6^	36	8%	64%	44%	5.9	3.7	9.5	8.0	5.0	11.6
		10^5^	20	30%	30%	80%	9.5	4.7	11.4	12.1	6.5	13.0
		10^3^	20	65%	5%	40%	9.4	6.0	9.9	9.9	6.0	10.8
		10^2^	10	100%	0%	0%	…	…	…	…	…	…
	IP	10^6^	15	7%	0%	87%	8.3	…	8.3	8.6	…	8.3
		10^5^	10	20%	0%	80%	9.4	…	9.9	9.8	…	10.3
		10^4^	48	19%	21%	75%	8.6	7.4	9.1	9.6	8.0	10.0
		10^3^	15	47%	0%	53%	12.4	…	12.4	13.5	…	13.5

Groups of Syrian hamsters were inoculated with either Nipah-Malaysia or Nipah-Bangladesh via the intranasal (IN) or intraperitoneal (IP) route, with doses ranging from 10^2^ to 10^7^ tissue culture infectious dose 50% (TCID_50_). Survival, incidence of respiratory (R) and/or neurological (N) clinical signs, mean-day-onset (MDO), and mean-time-to-death (MTTD) are indicated. Analyses include animals originally designated as terminal study groups.

Overall, individual hamsters exhibited the following clinical presentations over the observation period: (i) no clinical signs (14.4% [50/348]); (ii) respiratory signs only (19.5% [68/348]); (iii) neurological signs only (42.5% [148/348]); (iv) concurrent respiratory and neurological signs (4.3% [15/348]); or (v) respiratory signs followed by neurological signs (12.4% [43/348]). Assessing longitudinal individual data, IN-inoculated hamsters were more likely to exhibit a biphasic phenotype (i.e., respiratory signs followed by neurological signs) than IP cohorts. This phenotype was seen in 37% of hamsters with neurological signs inoculated with NiV-M IN versus only 16% of those IP-inoculated, and in 25% of hamsters with neurological signs IN-inoculated with NiV-B versus only 5% of those IP-inoculated (Supplementary Table 3).

### Clinical Outcome May be Affected by Age but not by Sex in the Syrian Hamster Model of NiV

**Figure 2.**
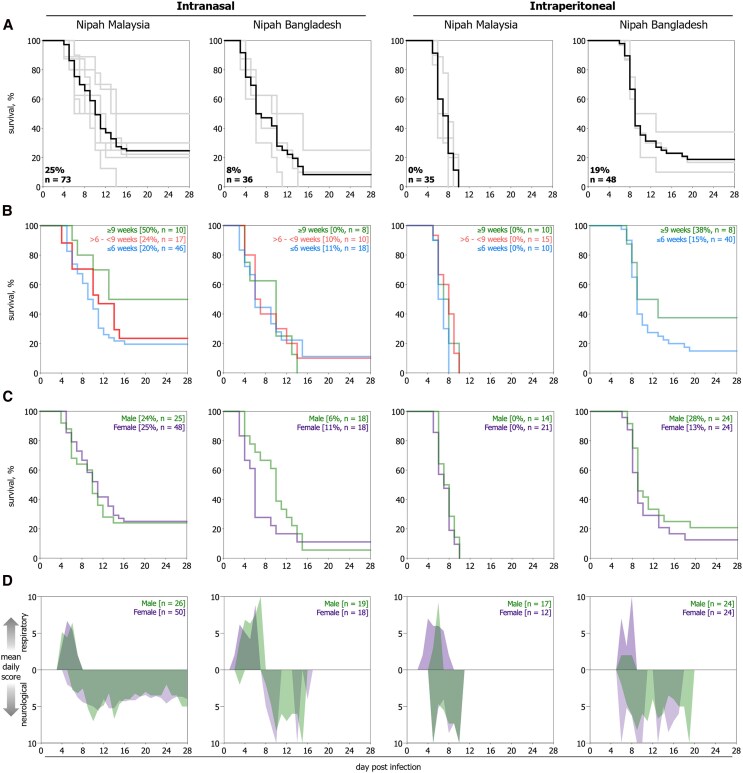
Age but not sex may be associated with differences in outcome in Syrian hamster models of Nipah virus infection. Kaplan–Meier survival curves of Syrian hamsters inoculated with NiV Malaysia or NiV Bangladesh either intranasally (10^6^ TCID_50_) or intraperitoneally (10^4^ TCID_50_). *A*, Survival curves for each independent study conducted (gray), with the survival curves for each experimental cohort overall (black, comprising 3–8 independent studies). *B*, Survival curves for age-grouped hamsters. Age of hamsters on day of inoculation for each study is indicated: ≤ 6 weeks (34–42 days, blue), > 6 to < 9 weeks (45–62 days, red), ≥ 9 weeks (63–75 days, green). *C*, Survival curves comparing male (green) and female (purple) hamsters within each experimental cohort. *D*, Temporal analysis of clinical signs for male (green) and female (purple) cohorts of Syrian hamsters inoculated with NiV. Respiratory signs are indicated upward from the *x*-axis, and neurological signs are indicated downward. Clinical scores (1–10) are the average score of scoring individuals for each day until study end (28 days post-infection).

To confirm that an aggregate data analysis approach was appropriate and ensure subsequent analyses were not influenced by study-to-study variation, IPD meta-analyses were used to assess between-study variability and within-animal correlation over time. Significant disease progression was observed across all groups (*P* <.001). Variability in clinical severity was primarily attributable to differences between individual animals (SD = 0.87–3.83), with low between-study variance (SD = 0.0–0.6), except for NiV-M IP, where variation was similarly attributable between individual animals (SD = 1.95) and studies (SD = 1.52). Similarly, survival outcomes were consistent across most cohorts (between-study SD ≤ 0.6), while NiV-M IP exhibited higher study-level variance (SD = 1.4), likely reflecting the rapid lethality of this cohort. For the most represented cohorts in our dataset (NiV-M or NiV-B, either IN with 10^6^ TCID_50_, or IP with 10^4^ TCID_50_), matched experimental cohorts (3–8 studies per cohort) demonstrated comparable survival rates (Figure 2*A*, Supplementary Table 4), with no significant differences observed between individual study survival rates and the cohort average. When individual studies were compared directly, significant differences appeared in only 9% (4/43) of pairwise comparisons (Supplementary Table 5). Overall, these analyses support the robustness of the combined dataset and indicate that study-level differences contributed minimally to outcome variability for most cohorts.

Analyses of age- and sex-associated differences in outcome were performed using the same experimental cohorts. For age, hamsters were grouped by weeks of age at inoculation: ≤6 weeks (34–42 days), >6 to <9 weeks (45–62 days), ≥9 weeks (63–75 days). In cohorts with less severe outcomes overall, animals ≥9 weeks (n = 8–10) showed higher survival compared to those <9 weeks (n = 17–46); 50% versus 20%–24% for NiV-M IN and 38% versus 15% for NiV-B IP (Figure 2*B*, Supplementary Table 5), but neither difference reached statistical significance. For sex, we previously reported no associated differences in humoral immune responses to NiV in hamsters [26]. Here, comparing males (n = 17–24) and females (n = 18–44), we found no significant sex-based differences in outcomes by strain or route (Figure 2*C*, Supplementary Table 5), and males and females demonstrated similar clinical progression and severity (Supplementary Fig. 2D). Additionally, analysis of vRNA levels in tissue revealed no significant differences (Supplementary Table 6).

### Weight and Temperature as Indicators of Respiratory Disease Progression in Syrian Hamsters Inoculated Intranasally With NiV

Alterations in measurable physiological parameters, such as body weight and temperature, are valuable indicators of disease progression in rodent models. As rodents can mask clinical signs until illness becomes severe [27], we analyzed temporal patterns of objective measures (body weight and temperature) in NiV-infected hamster cohorts categorized by primary clinical presentation (i.e., predominantly respiratory or neurological clinical signs; Supplementary Fig. 1). We compared animals that succumbed to disease with those that survived to: (i) identify clinical trends; (ii) assess whether these trends were influenced by the route of inoculation; and (iii) evaluate the predictive value of these parameters for disease severity and outcome in Syrian hamsters. To minimize bias introduced by variability in disease onset and time-to-death among animals with neurological disease, we used Spearman nonparametric correlation analysis to assess associations between changes in clinical scores and weight or temperature.

**Figure 3.**
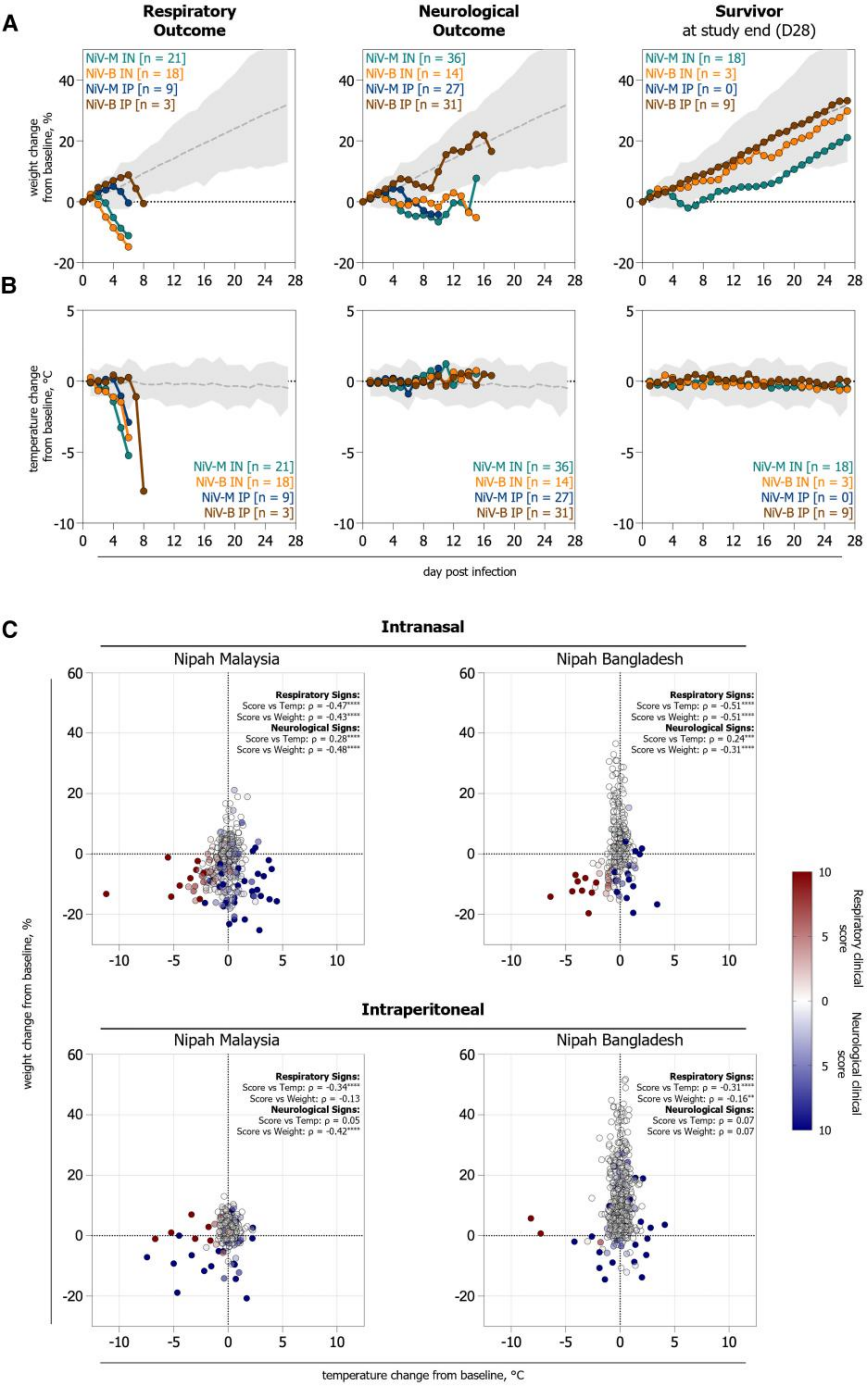
Fatal respiratory disease in Syrian hamsters following intranasal Nipah virus inoculation is associated with early temperature alterations and weight loss. Syrian hamsters were inoculated with NiV Malaysia (NiV-M) or NiV Bangladesh (NiV-B) either intranasally (IN, 10^6^ TCID_50_) or intraperitoneally (IP, 10^4^ TCID_50_). Average percentage weight change from baseline **(***A***)** and temperature change (°C) from baseline **(***B***)** for each cohort. Animals succumbing to respiratory outcome, neurological outcome, and survivors to study end (28 days post infection) are grouped. Any animals where endpoint could not be characterized were excluded from analysis. The average percentage weight change from baseline **(***A***)** and temperature change (°C) from baseline **(***B***)** for mock-inoculated (DMEM-only), untreated, unvaccinated control hamsters (n = 64) is indicated by the dotted line and the range indicated by the shaded area. *C*, Correlation analysis of daily percentage weight change from baseline, daily temperature change (°C) from baseline, and score severity (0–10) for each cohort was performed. Clinical sign is indicated by red (respiratory), blue (neurological), or white (no sign), with severity depicted by increased intensity. Spearman correlation analysis was performed on measurements collected longitudinally from individuals succumbing to terminal disease (NiV-M IN [n = 50], NiV-B IN [n = 32], NiV-M IP [n = 35], NiV-B IP [n = 34]), the correlation coefficient is indicated by “ρ”. Significance stated as; ****P ≤ .0001; ***P ≤ .001; **P ≤ .01; *P ≤ .05. Non-significance is not shown.

Animals succumbing to respiratory disease exhibited decreases in body weight and temperature from as early as 1 dpi (Figure 3*A*, 3*B*). All animals (n = 51) exhibiting a decrease in body temperature >2°C from their baseline value succumbed to disease. Animals IN-inoculated with either NiV strain that succumbed to respiratory disease demonstrated moderate correlation (Spearman correlation coefficient (*ρ*) ± 0.4–0.6) with increased clinical score, and decreased weight and body temperatures. Similarly, animals inoculated IN with NiV-M or NiV-B that succumbed to neurological disease demonstrated moderate correlation with increased clinical score and decreased weight only (Figure 3*C*). In contrast, animals IP-inoculated with NiV-M or NiV-B that succumbed to either terminal respiratory or neurological disease only demonstrated negligible (ρ±0.0–0.2) to weak (ρ±0.2–0.4) correlations between changes in weight and body temperatures with clinical score. The only exception was for hamsters IP-inoculated with NiV-M, where those who succumbed to neurological disease displayed a moderate correlation between increased disease severity and decreased weight.

### Temporal and Tissue-specific Viral RNA Levels Correlate With Disease Outcome and are Influenced by NiV Strain and Inoculation Route

**Figure 4.**
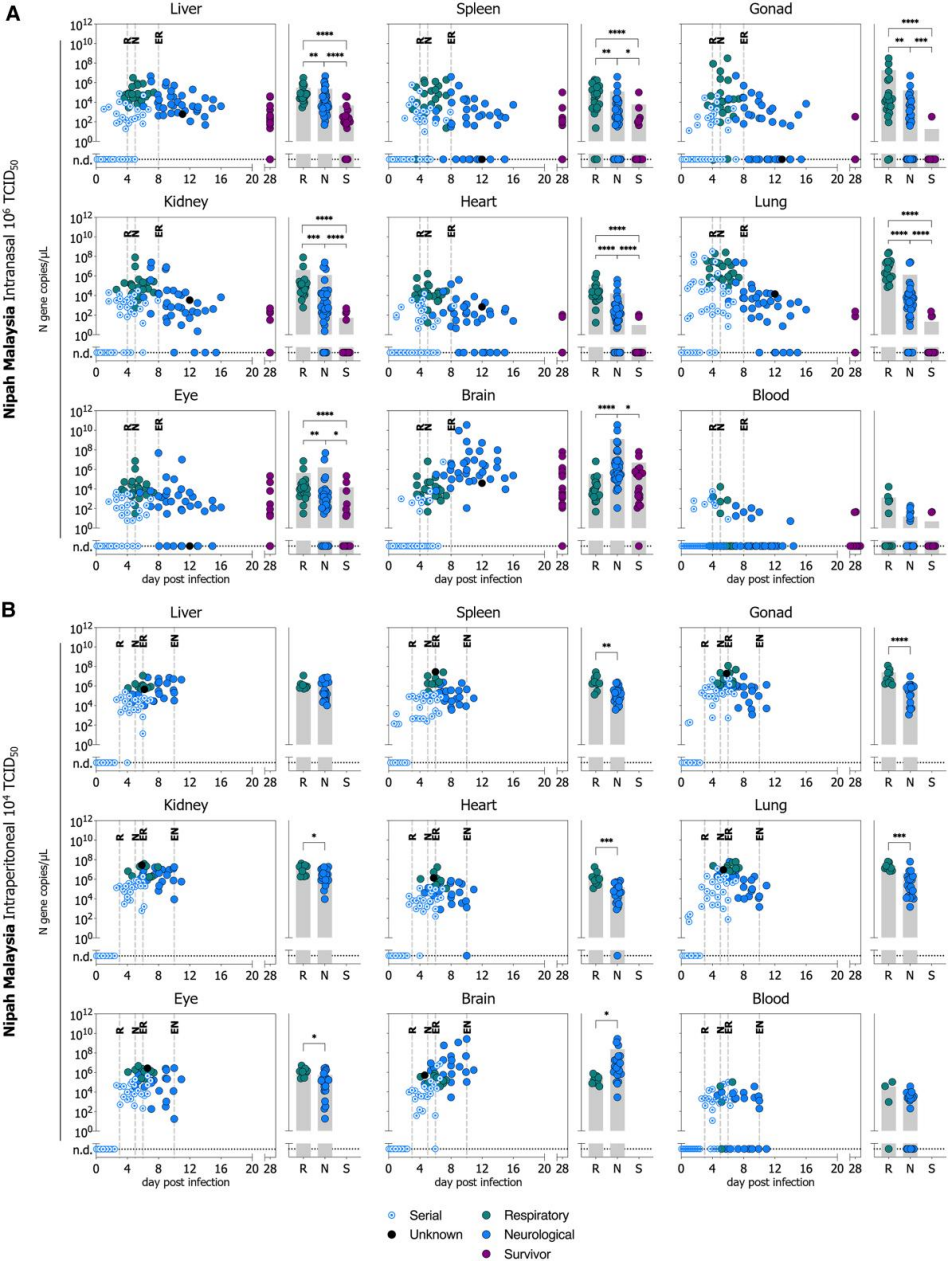
Temporal and outcome-associated viral RNA tissue levels in Syrian hamsters inoculated with Nipah virus strain Malaysia. RT-qPCR detection of NiV vRNA in select tissues (gonad denotes testis or ovary) from Syrian hamsters inoculated with NiV Malaysia **(***A***)** intranasally (10^6^ TCID_50_ [n = 115]) or **(***B***)** intraperitoneally (10^4^ TCID_50_ [n = 66]). Temporal data points include serial time points (1–7 days post-infection, blue circle with dot), respiratory outcome (teal circle, R), neurological outcome (blue circle, N), or survivors (purple circle, S), plotted against time-of-collection. Samples with an unknown cause of outcome are indicated with black circles. Vertical dashed lines indicate either the onset of respiratory (R) or neurological (N) signs, or the last occurrence (i.e., end) of respiratory (ER) or neurological (EN) signs. Horizontal dotted line indicates no viral RNA was detected (n.d.). Terminal outcome groups were combined, and gray bars indicated the mean of all samples at respiratory outcome, neurological outcome, and survivors. Significance was calculated for terminal outcome using the Mann-Whitney unpaired test using the Holm–Šídák method; ****P ≤ .0001; ***P ≤ .001; **P ≤ .01; *P ≤ .05. Non-significance is not shown.

**Figure 5.**
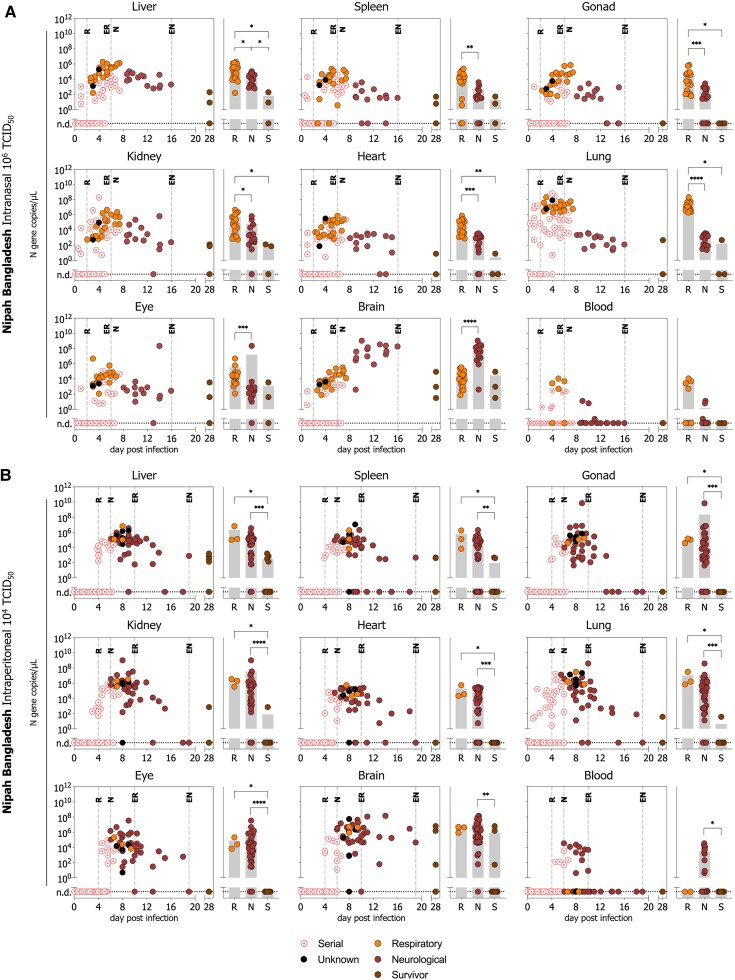
Temporal and outcome-associated viral RNA tissue levels in Syrian hamsters inoculated with Nipah virus strain Bangladesh. RT-qPCR detection of NiV vRNA in select tissues (gonad denotes testis or ovary) from Syrian hamsters inoculated with NiV Bangladesh **(***A***)** intranasally (10^6^ TCID_50_ [n = 76]) or **(***B***)** intraperitoneally (10^4^ TCID_50_ [n = 98]). Temporal data points include serial time points (1–6 days post-infection, pink circle with dot), respiratory outcome (orange circle, R), neurological outcome (dark red circle, N), or survivors (brown circle, S), plotted against time-of-collection. Vertical dashed lines indicate either onset of respiratory (R) or neurological (N) signs, or the last occurrence of respiratory (ER) or neurological (EN) signs. Horizontal dotted line indicates no viral RNA was detected (n.d.). Terminal outcome groups were combined, and gray bars indicated the mean of all samples at respiratory outcome, neurological outcome, and survivors. Significance was calculated for terminal outcome using the Mann-Whitney unpaired test using the Holm–Šídák method; ****P ≤ .0001; ***P ≤ .001; **P ≤ .01; *P ≤ .05. Non-significance is not shown.

To characterize viral biodistribution over time in experimentally infected Syrian hamsters, and inform the selection of meaningful endpoints for MCM evaluation, we quantified vRNA levels using RT-qPCR in liver, spleen, gonads (testis or ovary), kidney, heart, lung, eye, brain, and blood obtained from 355 hamsters that were serially euthanized between 1–7 dpi (n = 165), succumbed to terminal disease (n = 160; respiratory, neurological, unknown), or survived to study end at 28 dpi (n = 30) following inoculation IN with 10^6^ TCID_50_ or IP with 10^4^ TCID_50_ of NiV-M or NiV-B (Figures 4 and 5).

Viral RNA levels peaked early in infection (∼4–8 dpi) in all tissues except the brain, regardless of virus strain or inoculation route. At the respiratory disease endpoint, all cohorts had high vRNA levels in the lung (mean: 2.7×10^7^, range: 7.2×10^4^–2.6×10^8^ N gene copies/µL). Conversely, lung vRNA levels were significantly lower in animals succumbing to neurological disease, except in the NiV-B IP cohort, where the difference was not significant. At neurological disease endpoint, animals were likely to have higher vRNA levels in the brain compared to those succumbing to respiratory disease, with significant differences observed in IN-inoculated cohorts (both NiV-M and NiV-B). Survivors at study endpoint (28 dpi) had lower vRNA levels than animals that succumbed to terminal disease. However, in the NiV-M IN cohort, where 56% (10/18) of survivors exhibited ongoing neurological signs, high vRNA levels in the brain persisted. Notably, vRNA was detected in the brains of all survivors with persistent neurological signs.

We then compared tissue vRNA loads by strain (NiV-M vs NiV-B) and route of exposure (IN vs IP) again using samples from serially euthanized animals, terminal cases, and survivors (Supplementary Fig. 2A-B). In serial samples, IP inoculated NiV-M hamsters (our most lethal cohort) showed significantly higher vRNA levels in most tissues at 4 dpi compared to both NiV-M IN (route comparison) and NiV-B IP (strain comparison). In contrast, there were minimal differences by route for NiV-B or between strains for IN inoculation (Supplementary Fig. 2A). In terminal NiV-M hamsters, IP exposure led to higher tissue vRNA levels than IN (except lungs at respiratory outcome and brain at neurological outcome). NiV-B IP-inoculated hamsters generally demonstrated higher brain vRNA levels at the neurological endpoint. Notably, NiV-M and NiV-B yielded similar vRNA levels after IN inoculation, but after IP inoculation, NiV-M produced higher levels overall—except in the brain at the respiratory endpoint, where NiV-B was higher (Supplementary Fig. 2B).

## DISCUSSION

NiV infection in hamsters (Supplementary Table 7) recapitulates many of the clinical features reported in humans (Supplementary Table 8). Hamsters consistently develop respiratory disease and key neurological manifestations, including altered behaviors, impaired consciousness, locomotor dysfunction, paresis, paralysis, and seizures. Our data indicate that NiV infection in Syrian hamsters can be tailored by choice of strain, route, and dose to produce one of three outcomes: (i) respiratory and neurological disease, (ii) predominantly respiratory disease, or (iii) predominantly neurological disease. High-dose inoculation (10^6^ TCID_50_ IN or 10^4^ TCID_50_ IP) with either strain resulted in the full spectrum of disease. Respiratory disease was predominantly observed with the highest dose (10^7^ TCID_50_) of NiV-M administered IN, and neurological disease was predominantly observed via IP inoculation, particularly with NiV-B where respiratory signs were rarely observed (Supplementary Table 3).

Furthermore, these data can be used to guide studies aimed at increasing the proportion of symptomatic or asymptomatic survivors. This is particularly relevant for NiV, where neurological sequelae are well documented [28–31], and occur in both recrudescent and late-onset disease. Late-onset and relapsing neurological disease are thought to result from persistent infection in the brain, as observed in humans and primates [29, 32]. Like previous reports [9, 17–20, 22, 33], we found that infection in hamsters is not uniformly lethal. In the hamster model we observed a high proportion of survivors demonstrating neurological signs and vRNA in brain tissue at study end. Long-term neurological signs, characterized here as present at 25–28 dpi, while occasionally observed in NiV-B IN and IP, were common in NiV-M IN survivors (Figure 1*D*), indicating this model is well-suited to study persistent NiV-M infection.

We examined sex and age as potential factors influencing outcomes in the hamster model. Although sex [26, 34] and age [35] have been investigated in other high-consequence viral models, data remain limited. NiV studies have predominantly used female hamsters [11, 17–19 , 21 , 23 , 33, 36, 37] and younger cohorts (5–8 weeks), with no prior reports of sex- or age-based outcome differences. In our analyses, outcome did not differ by sex, though immune, hormonal, and metabolic factors were not assessed. Such variables can affect MCM responses, for example, male hamsters show greater pulmonary viral clearance than females after molnupiravir treatment in severe acute respiratory syndrome coronavirus 2 (SARS-CoV-2) studies [38], indicating the importance of including both sexes in pre-clinical assessment. Age-associated lethality has been reported in other viral models (West Nile, influenza A, polio, and chikungunya virus [39–42]). In our study, animals ≥9 weeks of age exhibited decreased lethality in some analyses, though not significantly. These findings support further study of age-related effects, particularly for vaccine and durability studies using older animals.

Currently, hamsters, ferrets, and African green monkeys (AGMs) represent the most well-established NiV disease models [43]. In Syrian hamsters, outcome and clinical presentation vary across studies, even when the same strain, dose, and route are used (Supplementary Table 2), and few investigations have directly compared NiV-strains. Early comparative work in hamsters suggested lower pathogenicity of NiV-B versus NiV-M, regardless of route [17]. In contrast, our data demonstrate that NiV-B is less pathogenic than NiV-M following IP inoculation but more pathogenic via the IN route. In ferrets, strain comparisons are more limited, but available reports indicate similarly high lethality with both strains after IN inoculation, with NiV-M associated with faster disease progression [44, 45]. Consistent with our findings that respiratory exposure favors relatively greater virulence of NiV-B, an AGM study using IN/intratracheal inoculation reported more severe respiratory disease and higher lethality, correlating with higher viral loads in the lungs [46].

We focused on IN and IP inoculation routes. Both produced systemic infection, with lungs as the most frequent early detection site, independent of route. Human exposure arises from contact with reservoir (*Pteropus spp.* bat) linked sources, such as domestic animals or contaminated date palm sap, with over half of cases from subsequent person-to-person transmission, likely via mucosal exposure [1]. Of the routes tested, IN best represents natural infection. Other routes, including foodborne [36] and aerosol [47], may alter viral kinetics, clinical signs, and outcome in hamsters, warranting similar analyses depending on intended application of the models.

Aggregating individual-level data across multiple experimental studies, resulted in larger group sizes than typically used in individual studies, providing important advantages for data analysis; however, several limitations remain. This work represents a retrospective, aggregate analysis spanning multiple independent studies with varying group sizes, time points, and outcome measures, therefore a formal a priori power calculation was not appropriate. While higher-level groupings with large sample sizes, (e.g., survival analyses and correlations between clinical score and weight or temperature), likely had sufficient power to detect small differences, further stratified sub-analyses (e.g., by dose, age, or endpoint) were likely powered to detect only large effects. Consequently, subtle biological differences may not have been captured in these analyses. As the data encompassed many independent studies conducted over an extended period, we accounted for interstudy variation, and although these effects appear limited, some residual influence cannot be excluded.

Well-characterized animal models are critical tools for understanding pathogenesis, developing MCMs, and supporting biodefense and public health preparedness for NiV infection. Here, we examine experimental design-associated patterns and variability by analyzing clinical course, signs, outcome, and viral biodistribution in NiV-infected Syrian hamsters using a large in-house dataset. These data provide advanced insight into virus replication kinetics and clinical effects, helping define model strengths and limitations to guide improved study design and better mimic human disease.

## Supplementary Material

jiaf549_Supplementary_Data

## Supplementary Data

Supplementary materials are available at *The Journal of Infectious Diseases* online (http://jid.oxfordjournals.org/). Supplementary materials consist of data provided by the author that are published to benefit the reader. The posted materials are not copyedited. The contents of all supplementary data are the sole responsibility of the authors. Questions or messages regarding errors should be addressed to the author.

## References

[1] Spengler JR, Lo MK, Welch SR, Spiropoulou CF. Henipaviruses: epidemiology, ecology, disease, and the development of vaccines and therapeutics. Clin Microbiol Rev 2025; 38:e0012823. 39714175 10.1128/cmr.00128-23 PMC11905374

[2] Nikolay B, Ribeiro dos Santos G, Lipsitch M, et al Assessing the feasibility of Nipah vaccine efficacy trials based on previous outbreaks in Bangladesh. Vaccine 2021; 39:5600–6. 34426025 10.1016/j.vaccine.2021.08.027

[3] U.S. Food and Drug Administration . Animal rule summary. Available at: https://www.fda.gov/emergency-preparedness-and-response/preparedness-research/animal-rule-summary. Accessed 17 October 2024.

[4] Dups J, Middleton D, Long F, Arkinstall R, Marsh GA, Wang L-F. Subclinical infection without encephalitis in mice following intranasal exposure to Nipah virus-Malaysia and Nipah virus-Bangladesh. Virol J 2014; 11:102. 24890603 10.1186/1743-422X-11-102 PMC4057804

[5] Dhondt KP, Mathieu C, Chalons M, et al Type I interferon signaling protects mice from lethal henipavirus infection. J Infect Dis 2013; 207:142–51. 23089589 10.1093/infdis/jis653 PMC7107294

[6] de Wit E, Munster VJ. Animal models of disease shed light on Nipah virus pathogenesis and transmission. J Pathol. 2015; 235:196–205. 25229234 10.1002/path.4444 PMC4268059

[7] Harcourt BH, Tamin A, Halpin K, et al Molecular characterization of the polymerase gene and genomic termini of Nipah virus. Virology 2001; 287:192–201. 11504554 10.1006/viro.2001.1026

[8] Harcourt BH, Lowe L, Tamin A, et al Genetic characterization of Nipah virus, Bangladesh, 2004. Emerg Infect Dis 2005; 11:1594–7. 16318702 10.3201/eid1110.050513 PMC3366751

[9] Welch SR, Spengler JR, Genzer SC, et al Single-dose mucosal replicon-particle vaccine protects against lethal Nipah virus infection up to 3 days after vaccination. Sci Adv 2023; 9:eadh4057. 37540755 10.1126/sciadv.adh4057 PMC10403222

[10] Welch SR, Spengler JR, Harmon JR, et al Defective interfering viral particle treatment reduces clinical signs and protects hamsters from lethal nipah virus disease. mBio 2022; 13:e0329421. 35297677 10.1128/mbio.03294-21 PMC9040845

[11] Lo MK, Spengler JR, Welch SR, et al Evaluation of a single-dose nucleoside-modified messenger RNA vaccine encoding hendra virus-soluble glycoprotein against lethal nipah virus challenge in Syrian hamsters. J Infect Dis 2020; 221:S493–8. 31751453 10.1093/infdis/jiz553 PMC7368163

[12] Lo MK, Jain S, Davies KA, et al Optimization of Bangladesh and Malaysian genotype recombinant reporter Nipah viruses for in vitro antiviral screening and in vivo disease modeling. Antiviral Res 2024; 231:106013. 39326503 10.1016/j.antiviral.2024.106013 PMC11772256

[13] Lo MK, Spengler JR, Krumpe LRH, et al Griffithsin inhibits Nipah virus entry and fusion and can protect Syrian golden hamsters from lethal nipah virus challenge. J Infect Dis 2020; 221:S480–92. 32037447 10.1093/infdis/jiz630 PMC7199786

[14] Genzer SC, Welch SR, Scholte FEM, et al Alterations in blood chemistry levels associated with nipah virus disease in the Syrian hamster model. J Infect Dis 2020; 221:S454–9. 31747016 10.1093/infdis/jiz552 PMC7368165

[15] Patel K, Klena J, Lo MK. A revised diagnostic quantitative RT-PCR for the detection of Nipah virus infection. Methods Mol Biol 2023; 2682:25–31. 37610571 10.1007/978-1-0716-3283-3_2

[16] Davies KA, Welch SR, Sorvillo TE, et al Optimal reference genes for RNA tissue analysis in small animal models of hemorrhagic fever viruses. Sci Rep 2023; 13:19384. 37938597 10.1038/s41598-023-45740-w PMC10632498

[17] DeBuysscher BL, de Wit E, Munster VJ, Scott D, Feldmann H, Prescott J. Comparison of the pathogenicity of Nipah virus isolates from Bangladesh and Malaysia in the Syrian hamster. PLoS Negl Trop Dis 2013; 7:e2024. 23342177 10.1371/journal.pntd.0002024 PMC3547834

[18] Griffin BD, Leung A, Chan M, et al Establishment of an RNA polymerase II-driven reverse genetics system for Nipah virus strains from Malaysia and Bangladesh. Sci Rep 2019; 9:11171. 31371748 10.1038/s41598-019-47549-y PMC6671980

[19] de Wit E, Bushmaker T, Scott D, Feldmann H, Munster VJ. Nipah virus transmission in a hamster model. PLoS Negl Trop Dis 2011; 5:e1432. 22180802 10.1371/journal.pntd.0001432 PMC3236726

[20] Wong KT, Grosjean I, Brisson C, et al A golden hamster model for human acute Nipah virus infection. Am J Pathol 2003; 163:2127–37. 14578210 10.1016/S0002-9440(10)63569-9 PMC1892425

[21] Johnston SC, Qiu J, Norris SLW, et al Dose response comparison of Nipah virus strains Malaysia and Bangladesh in hamsters exposed by the intranasal or intraperitoneal route. PLoS One 2025; 20(5 MAY):e0318912. 40354368 10.1371/journal.pone.0318912 PMC12068590

[22] Findlay-Wilson S, Flett L, Salguero FJ, et al Establishment of a Nipah virus disease model in hamsters, including a comparison of intranasal and intraperitoneal routes of challenge. Pathogens 2023; 12:976. 37623936 10.3390/pathogens12080976 PMC10458503

[23] Lo MK, Bird BH, Chattopadhyay A, et al Single-dose replication-defective VSV-based Nipah virus vaccines provide protection from lethal challenge in Syrian hamsters. Antiviral Res 2014; 101:26–9. 24184127 10.1016/j.antiviral.2013.10.012 PMC3874889

[24] Ploquin A, Szécsi J, Mathieu C, et al Protection against henipavirus infection by use of recombinant adeno-associated virus-vector vaccines. J Infect Dis 2013; 207:469–78. 23175762 10.1093/infdis/jis699 PMC7107322

[25] Mathieu C, Guillaume V, Volchkova VA, et al Nonstructural Nipah virus C protein regulates both the early host proinflammatory response and viral virulence. J Virol 2012; 86:10766–75. 22837207 10.1128/JVI.01203-12 PMC3457280

[26] Scholte FEM, Rodriguez SE, Welch SR, et al Characterization of humoral responses to nipah virus infection in the Syrian hamster model of disease. J Infect Dis 2024; 230:438–43. 38064677 10.1093/infdis/jiad557 PMC11875002

[27] Frohlich J . Rodents—Exotic and Laboratory Animals. Merck Veterinary Manual. Available at: https://www.merckvetmanual.com/exotic-and-laboratory-animals/rodents/rodents. Accessed 16 March 2025.

[28] Goh KJ, Tan CT, Chew NK, et al Clinical features of Nipah virus encephalitis among pig farmers in Malaysia. N Engl J Med 2000; 342:1229–35. 10781618 10.1056/NEJM200004273421701

[29] Tan CT, Goh KJ, Wong KT, et al Relapsed and late-onset Nipah encephalitis. Ann Neurol 2002; 51:703–8. 12112075 10.1002/ana.10212

[30] Lam SK, Chua KB. Nipah virus encephalitis outbreak in Malaysia. Clin Infect Dis 2002; 34:S48–51. 11938496 10.1086/338818

[31] Sejvar JJ, Hossain J, Sana SK, et al Long-term neurological and functional outcome in Nipah virus infection. Ann Neurol 2007; 62:235–42. 17696217 10.1002/ana.21178

[32] Liu J, Coffin KM, Johnston SC, et al Nipah virus persists in the brains of nonhuman primate survivors. JCI Insight 2019; 4:e129629. 31341108 10.1172/jci.insight.129629 PMC6675545

[33] Freiberg AN, Worthy MN, Lee B, Holbrook MR. Combined chloroquine and ribavirin treatment does not prevent death in a hamster model of Nipah and Hendra virus infection. J Gen Virol 2010; 91:765–72. 19889926 10.1099/vir.0.017269-0 PMC2888097

[34] Klein S . Host factors mediating sex differences in viral infection. Gend Med. Elsevier 2005; 2:197–207. 10.1016/s1550-8579(05)80050-6 16464732

[35] Sorvillo TE, Ritter JM, Welch SR, et al Inflammation associated with monocyte/macrophage activation and recruitment corresponds with lethal outcome in a mouse model of crimean-Congo haemorrhagic fever1. Emerg Microbes Infect 2024; 13:2427782. 39513496 10.1080/22221751.2024.2427782 PMC11578417

[36] de Wit E, Prescott J, Falzarano D, et al Foodborne transmission of Nipah virus in Syrian hamsters. PLoS Pathog 2014; 10:e1004001. 24626480 10.1371/journal.ppat.1004001 PMC3953481

[37] Rockx B, Brining D, Kramer J, et al Clinical outcome of henipavirus infection in hamsters is determined by the route and dose of infection. J Virol 2011; 85:7658–71. 21593160 10.1128/JVI.00473-11 PMC3147900

[38] Lieber CM, Cox RM, Sourimant J, et al SARS-CoV-2 VOC type and biological sex affect molnupiravir efficacy in severe COVID-19 dwarf hamster model. Nat Commun 2022; 13:4416. 35906230 10.1038/s41467-022-32045-1 PMC9338273

[39] Crotty S, Hix L, Sigal LJ, Andino R. Poliovirus pathogenesis in a new poliovirus receptor transgenic mouse model: age-dependent paralysis and a mucosal route of infection. J Gen Virol 2002; 83:1707–20. 12075090 10.1099/0022-1317-83-7-1707

[40] Morrey JD, Day CW, Julander JG, et al Modeling hamsters for evaluating West Nile virus therapies. Antiviral Res 2004; 63:41–50. 15196819 10.1016/j.antiviral.2004.02.005

[41] Couderc T, Chrétien F, Schilte C, et al A mouse model for Chikungunya: young age and inefficient type-I interferon signaling are risk factors for severe disease. PLoS Pathog 2008; 4:e29. 18282093 10.1371/journal.ppat.0040029 PMC2242832

[42] Lu J, Duan X, Zhao W, et al Aged mice are more resistant to influenza virus infection due to reduced inflammation and lung pathology. Aging Dis 2018; 9:358–73. 29896425 10.14336/AD.2017.0701 PMC5988592

[43] Pigeaud DD, Geisbert TW, Woolsey C. Animal models for henipavirus research. Viruses 2023; 15:1980. 37896758 10.3390/v15101980 PMC10610982

[44] Leon AJ, Borisevich V, Boroumand N, et al Host gene expression profiles in ferrets infected with genetically distinct henipavirus strains. PLoS Negl Trop Dis 2018; 12:e0006343. 29538374 10.1371/journal.pntd.0006343 PMC5868854

[45] Clayton BA, Middleton D, Bergfeld J, et al Transmission routes for Nipah virus from Malaysia and Bangladesh. Emerg Infect Dis 2012; 18:1983–93. 23171621 10.3201/eid1812.120875 PMC3557903

[46] Mire CE, Satterfield BA, Geisbert JB, et al Pathogenic differences between Nipah virus Bangladesh and Malaysia strains in primates: implications for antibody therapy. Sci Rep 2016; 6:30916. 27484128 10.1038/srep30916 PMC4971471

[47] Escaffre O, Hill T, Ikegami T, et al Experimental infection of Syrian hamsters with aerosolized Nipah virus. J Infect Dis 2018; 218:1602–10. 29912426 10.1093/infdis/jiy357 PMC6173575

