# Supplementary material for: Natural History of Nipah Virus in Hamsters: Strain, Route, and Sex-Associated Variability Characterized Using Large Datasets to Inform Pre-Clinical Study Design
Source: J Infect Dis. 2025 Oct 29;233(2):e441–53. doi: 10.1093/infdis/jiaf549 (PMC13016801; doi:10.1093/infdis/jiaf549)
Supplement: jiaf549_Supplementary_Data [file jiaf549_supplementary_data.zip › v1_resub_JID_Davies_NiVAggregateAnalysis_SupMaterial.docx]

**Supplementary Materials**

**Natural History of Nipah Virus in Hamsters: Strain, Route, and Sex-Associated Variability Characterized using Large Datasets to Inform Pre-Clinical Study Design**

Katherine A. Davies^1,2^, Stephen R. Welch^1^, JoAnn D. Coleman-McCray^1^, Teresa E. Sorvillo^1,3^, Virginia Aida-Ficken^1,4^, Christina F. Spiropoulou^1^, Jessica R. Spengler^1*^

^1^Viral Special Pathogens Branch, Division of High-Consequence Pathogens and Pathology, Centers for Disease Control and Prevention, Atlanta, Georgia, USA

^2^Zoonotic and Emerging Disease Research Unit, National Bio and Agro-Defense Facility, Agricultural Research Service, United States Department of Agriculture, Manhattan, Kansas, USA

^3^Infectious Disease Department, CDC Foundation, Atlanta, GA, USA

^4^Department of Pathobiology, College of Veterinary Medicine, Auburn University, Auburn, Alabama, USA

*Corresponding author: Jessica R. Spengler; [wsk7@cdc.gov](mailto:wsk7@cdc.gov)

- **Supplementary materials and methods**
- **Supplementary figures**
  - **Supplementary Figure 1. Temperature change and weight loss as markers of clinical disease in NiV-infected Syrian hamsters.**
  - **Supplementary Figure 2. Viral RNA tissue loads are altered depending on Nipah virus strain and inoculation route.**

**Materials and methods**

***Animal Housing***

Hamsters were group-housed in a climate-controlled laboratory (68–79°C and 30–70% humidity) with a 12-hour day/night cycle, on corn cob bedding (Bed-o’Cobs ¼″, Anderson Lab Bedding) with cotton nestlets and crinkle paper (Enviro-dri, Shepherd Specialty Papers) in isolator-caging systems (Thoren Caging Size 6 or Tecniplast GR900), and provided Teklad global 18% protein rodent diet (Envigo) and water *ad libitum*. Microchip transponders (Bio Medic Data Systems, IPTT-300) were placed subcutaneously in the interscapular region for identification and to assess body temperature, and insertion sites closed with GLUture topical tissue adhesive (MWI Veterinary).

***Clinical Scoring***

Syrian hamsters were assessed daily for clinical signs, weight change, and temperature. Baseline weights were taken at either -1 or 0 days post-infection (dpi). Body temperature was recorded daily, and percentage change was calculated relative to baseline. Baseline temperature was defined as the average over 2–3 days beginning on or before inoculation and prior to onset of clinical signs (i.e., from readings taken between -1 to 2 dpi). Clinical signs were quantified as 2 points each for mild signs (generic: quiet, dull, hunched back, ruffled coat, hypoactivity, squint, grimace, respiratory: mild increase in respiratory rate and effort, neurological: <30° head tilt, dystaxia, abnormal gait, overgrooming), 5 points each for moderate signs (generic: hypothermia (<34°C or >2°C decrease from baseline), respiratory: moderate increase in respiratory rate and effort, labored breathing, tucked waist, neurological: <30-90° head tilt, circling, ataxia, tremors, paresis, aggressive behavior, reactive behavior, absent seizure), and 10 points each for severe signs (generic: moribund, >25% weight loss from baseline), respiratory: severe increase in respiratory rate and effort, frank hemorrhage, neurological: >90° head tilt, paralysis, inability to right self, clonic seizure, unresponsive, unable to obtain food and water) (Fig. 1B).

***RNA Extraction***

RNA was extracted with the MagMAX-96 Total RNA isolation kit or MagMAX Pathogen RNA/DNA Kit using the KingFisher Flex System or KingFisher Apex System (all Thermo Fisher Scientific). For tissue analysis, small sections of liver, spleen, gonad (testes or ovary), kidney, heart, lung, eye, and brain were homogenized in MagMAX Lysis/Binding Solution Concentrate. Whole blood was collected in lithium heparin or potassium EDTA microtubes or vacutainers before adding 50 µL whole blood to 500 µL MagMAX Lysis/Binding Solution Concentrate. RNA was extracted from 250 µL lysate, and 150 µL isopropanol added at time of extraction. Samples were treated with either Turbo-DNase (Thermo Fisher) or DNase-1 (Biosearch Technologies) and eluted in 75 µL elution buffer. RNA was stored at -80°C before analysis.

***Real-Time Quantitative PCR***

Viral RNA in tissues was quantified by RT-qPCR targeting the NiV nucleoprotein (NP) [15]. Primer sequences (5ʹ-3ʹ) were as follows: (NiV forward (400nM), CTG GTC TCT GCA GTT ATC ACC ATC GA; NiV reverse (400 nM), ACG TAC TTA GCC CAT CTT CTA GTT TCA; and NiV probe (200 nM), (6-FAM)-CAG CTC CCG -(ZEN) - ACA CTG CCG AGG AT-(IBFQ). Levels of viral RNA were standardized using in-house validated reference gene RT-qPCR assays for Peptidylprolyl isomerase A (*Ppia*) and Hypoxanthine phosphoribosyl transferase (*Hprt*) [16], or using a commercial Eukaryotic 18s rRNA Endogenous Control assay (Thermo Fisher Scientific). Primer sequences for the in-house assay were as follows: Pan-rodent *Ppia* forward (400 nM); CCC ACC GTG TTC TTC GAC, Pan-rodent *Ppia* reverse (400 nM); TCC TTT CTC TCC AGT GCT CAG, Pan-rodent *Ppia* probe (200 nM); (HEX)-CCT TGG GCC - (ZEN) - GCG TCT CCT TCG A-(IBFQ), Hamster *Hprt* forward (550 nM); AGC CTG TTG GGC TTA CTT CC, Hamster *Hprt* reverse (400 nM); ATC ACG ACG CTG GGA CTG; Hamster *Hprt* probe (450 nM); (Cy5)-ACC GAT TCC - (TAO) - GTC ATG GCG AC-(IBRQ). All RT-qPCRs were performed using the SuperScript III Platinum One-Step RT-qPCR kit (Thermo Fisher Scientific). Genome copies per µL extract were calculated against a standard curve of synthetic RNA diluted to known copy numbers. All in-house assay primers, probes, and synthetic RNAs were synthesized by Integrated DNA Technologies (IDT).

**
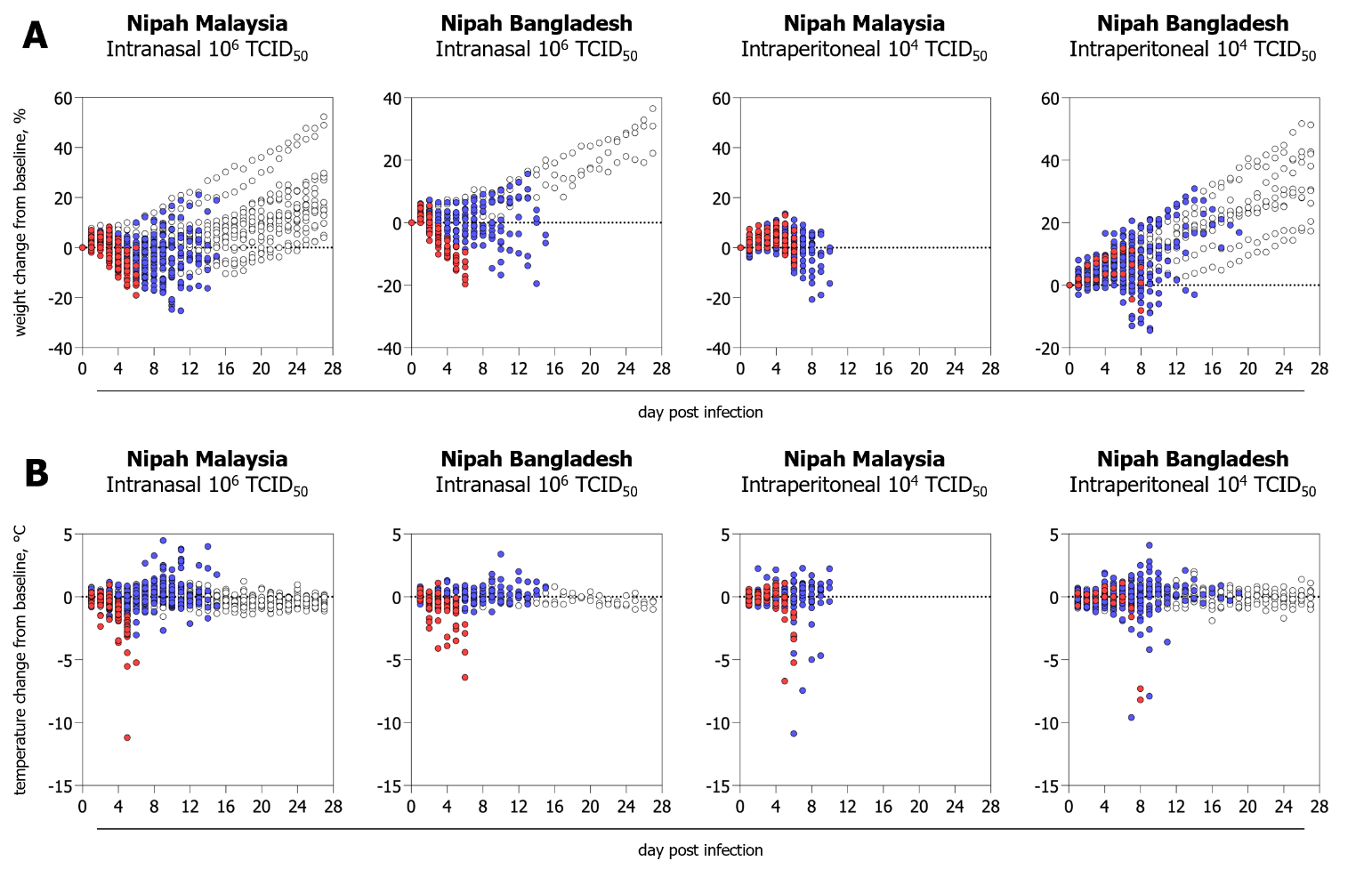
**

**Supplementary Figure 1. Temperature change and weight loss as markers of clinical disease in NiV-infected Syrian hamsters.** Syrian hamsters challenged with NiV Malaysia (NiV-M) or Bangladesh (NiV-B) either intranasal (IN, 10^6^ TCID_50_) or intraperitoneally (IP, 10^4^ TCID_50_). Temperature change (°C) from baseline (A) and percentage weight change from baseline (B) for individuals within each cohort across infection course; NiV-M IN [n = 76], NiV-B IN [n = 41], NiV-M IP [n = 37], NiV-B IP [n = 48]. Data point color indicate outcome; respiratory (red), neurological (blue) and survivor (white).


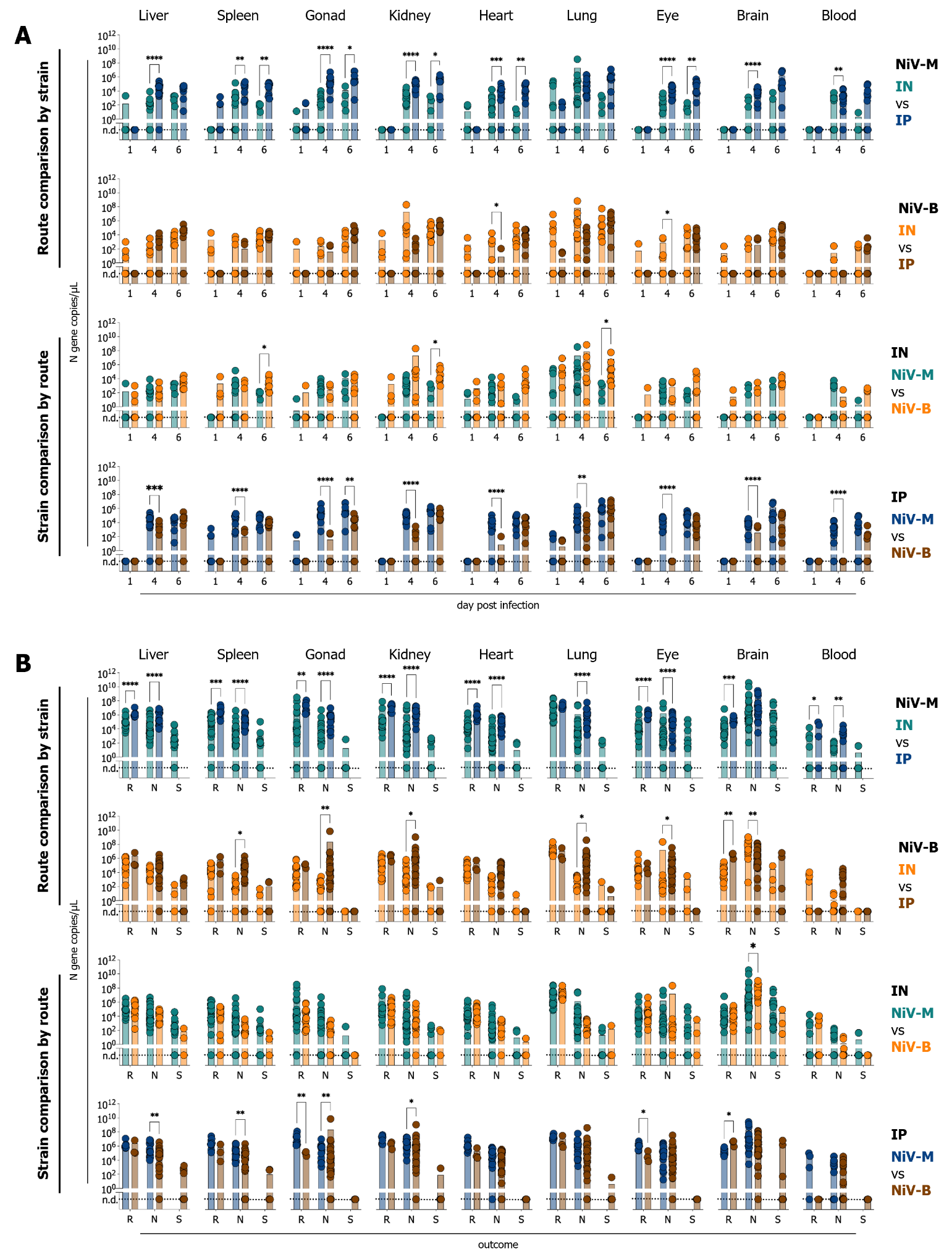


**Supplementary Figure 2. Viral RNA tissue loads are altered depending on Nipah virus strain and inoculation route.** RT-qPCR detection of NiV vRNA in select tissues from Syrian hamsters inoculated intranasally (IN, 10^6^ TCID_50_) or intraperitoneally (IP, 10^4^ TCID_50_) with NiV Malaysia (NiV-M) or Bangladesh (NiV-B). Comparisons between (A) samples collected at serial time points (1-, 4- and 6-days post infection) (NiV-M IN [n = 13, 17, 4], NiV-B IN [n = 15, 10, 9], NiV-M IP [n = 14, 14, 9], NiV-B IP [n = 15, 15, 10]) and (B) samples collected at respiratory (R) outcome (NiV-M IN [n = 21], NiV-B IN [n = 18], NiV-M IP [n = 9], NiV-B IP [n = 3]), neurological (N) outcome (NiV-M IN [n = 36], NiV-B IN [n = 14], NiV-M IP [n = 20], NiV-B IP [n = 31]), and survivors (S) (NiV-M IN [n = 18], NiV-B IN [n = 3], NiV-M IP [n = 0], NiV-B IP [n =9]). Analyses are conducted comparing N gene copies/µL either by strain or by route.‘n.d.’ indicates no viral RNA was detected. Significance was calculated using the Mann-Whitney unpaired test using the Holm-Šídák method; ****p ≤ 0.0001; ***p ≤ 0.001; **p ≤ 0.01; *p ≤ 0.05. Non-significance is not shown.
